# Temporal intensity correlation of bunched light from a warm atomic vapor with a ladder-type two-photon transition

**DOI:** 10.1038/s41598-018-29340-7

**Published:** 2018-07-20

**Authors:** Jiho Park, Taek Jeong, Han Seb Moon

**Affiliations:** Department of Physics, Pusan National University, Geumjeong-Gu, Busan, 46241 South Korea

## Abstract

We report the temporal intensity correlation (TIC) of scattered photons (SPs) generated via a two-photon transition in a Doppler-broadened warm atomic vapor of the 5S_1/2_ − 5P_3/2_ − 5D_5/2_ transition of ^87^Rb atoms. Through the investigation of the TICs of the SPs obtained via both one- and two-photon transitions, the second-order correlation values *g*^(2)^(0) (i.e., at zero time delay) of both SPs were measured as approximately 1.75, respectively. The widths of the *g*^(2)^(*τ*) spectra were measured as 26 ns (corresponding to the natural lifetime of the 5P_3/2_ state) for the one-photon transition and 1.8 ns (corresponding to the Doppler width of the warm atomic vapor) for the two-photon transition. We confirmed that the coherence time of the SPs can vary in accordance with the photons emitted from the one- or two-photon transitions in the ladder-type atomic system. The correlated SPs obtained via the two-photon transition contributed to almost all the velocity classes of the atoms in the Doppler-broadened atomic ensemble.

## Introduction

Light is understood in terms of fundamental concepts related to both the wave property and particle property, such as optical coherence and photon statistics, respectively^1^. Natural light is well known to constitute thermal light. Further, thermal light in an electromagnetic wave corresponds to incoherent light, which is described by the superposition of many waves with random amplitudes and phases^2^. However, in the photon context, thermal light is understood as a bunching of photons and examined in terms of the temporal statistical properties of those photons. Recently, in quantum optics, the bunching properties of thermal light were employed in imaging and interference experiments to simulate entangled states^3–7^.

The difficulty in measuring the temporal statistical properties of natural light beam is well known, which is due to the lack of detector time resolution and imperfect spatial coherence in the measurements. However, the fluorescence emitted from the atoms has a longer coherence time than the detector time resolution. The second-order temporal correlation has been measured in various atomic media, such as atomic beams^8^, discharge lamps^9^, cold atoms^10–13^, and warm atomic vapor cells^14^. Although the second-order temporal correlation of the fluorescence from various atomic media has been definitively observed as photon bunching at zero time delay, the spectral features in that case are dependent on the characteristics of the atomic media, such as multiscattering, Doppler broadening, and homogenous effects^8–14^.

However, a two-photon transition of a ladder-type atomic system, which is generated by the interaction of an atom with two coherent electromagnetic fields, exhibits various two-photon coherence phenomena^15–22^. In quantum optics, the high-performance generation of correlated photon pairs in a ladder-type atomic system via the spontaneous four-wave mixing (SFWM) process has recently been reported^23–27^. Interestingly, in spite of thermal atomic motion, the efficient generation of heralded single photons from the collective excitations created in warm atomic vapor has been experimentally demonstrated^28,29^. In addition, studies on quantum memory and quantum interference using a Doppler-broadened ladder-type atomic medium have been reported^30,31^. However, although the properties of the photons emitted from a ladder-type atomic system are important, the dependence of the temporal statistical properties of the obtained photons on the one- or two-photon transitions in a ladder-type atomic system have not been studied.

We herein investigate the temporal statistical properties of these two kinds of scattered photons (SPs), i.e., those resulting from one- and two-photon transitions, from a Doppler-broadened warm atomic vapor, and from the 5S_1/2_ − 5P_3/2_ − 5D_5/2_ transition of ^87^Rb atoms. Using the Hanbury-Brown-Twiss (HBT) experiment^32^, we examine the dependence of the temporal intensity correlation (TIC) spectrum of the photons emitted from the warm ^87^Rb vapor cell on the one-photon resonance of the 5S_1/2_ − 5P_3/2_ transition and the two-photon resonance of the 5S_1/2_ − 5P_3/2_ − 5D_5/2_ transition. In addition, we investigate the variance of the temporal statistical properties of the SPs according to the detuning frequencies of the lasers used to generate an excited state. The spectral features of the TIC are elucidated by examining the two-photon resonance contributions from almost all the atomic velocity groups in the Doppler-broadened ladder-type atomic system.

## Experimental Setup

**Figure 1:**
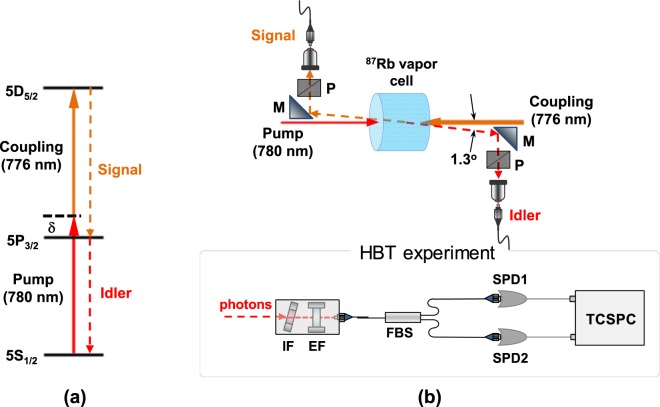
Photon-pair generation in ladder-type atomic system. (**a**) Energy-level diagram of ladder-type 5S_1/2_ − 5P_3/2_ − 5D_5/2_ transition of ^87^Rb atoms. (**b**) Experimental schematic for photon-pair generation in ^87^Rb atomic vapor cell and Hanbury Brown-Twiss (HBT) experimental setup for obtaining normalized auto-correlation functions for individual signal and idler photons (P: polarizer; M: mirror; IF: interference filter, EF: solid fused-silica etalon filter; FBS: fiber beam splitter; single-photon detectors (SPDs); TCSPC: time-correlated single-photon counting module).

As shown in Fig. 1(a), this ladder-type atomic system consists of a ground state (5S_1/2_), an intermediate state (5P_3/2_), and an excited state (5D_5/2_). The natural linewidths of the 5P_3/2_ and 5D_5/2_ states are 6.1 MHz and 0.67 MHz, respectively. In this experiment, the pump and coupling lasers were coupled with the 5S_1/2_ − 5P_3/2_ and 5P_3/2_ − 5D_5/2_ transitions, respectively. To satisfy the condition for the two-photon resonance between both lasers, the frequencies of the pump and coupling lasers were detuned to red and blue shifts of *δ* from the resonance of the 5S_1/2_ − 5P_3/2_ and the 5P_3/2_ − 5D_5/2_ transitions, respectively.

Figure 1(b) is a schematic of the experimental setup used to obtain the Doppler-free two-photon resonance in the Doppler-broadened ladder-type atomic system. A rubidium vapor cell contained the enriched ^87^Rb atoms, having a diameter of 25 mm and a thickness of 12.5 mm. Here, the pump and coupling lasers were counter-propagated through a ^87^Rb vapor cell. Two laser beams with beam diameters of 1.2 mm were completely and spatially overlapped. The polarizations of the pump and coupling lasers were linearly polarized and perpendicular. Two external cavity diode lasers (ECDLs) were operated independently at wavelengths of 780.2 nm (pump) and 775.8 nm (coupling). The vapor-cell temperature was set to 80 °C. The signal and idler photons were spontaneously emitted from the vapor cell under the condition of two-photon resonance in the phase-matched direction. To separate the two lasers, the signal and idler photons were collected at a tilted angle (*θ*_t_) of 1.3° in two single-mode fibers (SMFs) at a distance of 50 cm from the vapor cell.

To investigate the temporal statistical properties of the SPs, the HBT experimental setup is used to investigate the photon statistical property, which utilized the spontaneously emitted signal and idler photons. The schematic of the setup is shown in Fig. 1(b). We used the polarizers and etalon filters to remove the scattered laser component and uncorrelated fluorescence. In particular, the etalon filters have a full width at half maximum (FWHM) linewidth of 950 MHz, free spectral range of 20 GHz, and 90% peak transmission. We can select the Rayleigh SPs of the 5S_1/2_(F = 2) state − 5P_3/2_ (F′ = 1, 2, 3) transition and remove the Raman SPs of the 5S_1/2_(F = 1) state − 5P_3/2_ (F′ = 1, 2) transition. From the measured results of the HBT experiment, we estimated the normalized second-order auto-correlation functions *g*^(2)^(*τ*) for photons in both the signal and idler modes, where *τ* is the time delay between two photon counting events measured using two SPDs. The time uncertainties of the two SPDs were measured as 0.42 ± 0.05 ns and 0.46 ± 0.05 ns, respectively, including the electronic timing jitter generated when using a weak mode-locked picosecond-pulse laser. The collected signal or idler photons has passed through a 50/50 fiber beam splitter and were detected by the two SPDs. A histogram of the coincidence events between SPD1 and SPD2 as a function of *τ* was obtained using a time-correlated single-photon counter (TCSPC) in the start-stop mode with a 32-ps time resolution.

## Results

In quantum optics, the most popular photon-pair source is the correlated photon pairs generated via the spontaneous parametric down-conversion (SPDC) process in χ^(2)^ nonlinear crystals^33–35^. However, their coherence time is shorter than the response time of commercial single-photon detectors (SPDs). Although the photons in each mode of the correlated photon pairs exhibit the photon statistical property of thermal light, it is difficult to observe the photon bunching effect in the individual modes of the SPDC sources except for the cavity-enhanced SPDC^36,37^. In contrast, however, the photons produced from a warm atomic vapor of ^87^Rb atoms have a relatively long coherence time. We experimentally demonstrated the TIC of bunched light from a warm atomic vapor obtained via a ladder-type two-photon transition, i.e., the 5S_1/2_ − 5P_3/2_ − 5D_5/2_ transition of ^87^Rb.

### Scattered photons via one-photon transition in Doppler-broadened atomic medium

**Figure 2:**
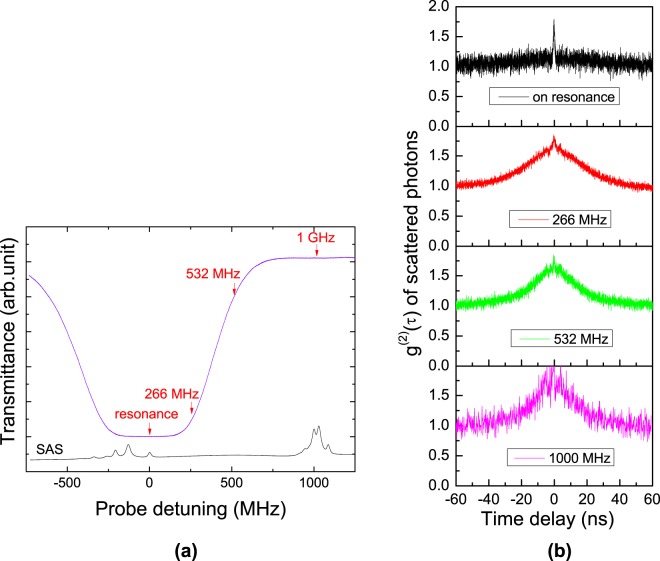
Scattered photons via one-photon transition in Doppler-broadened atomic medium. (**a**) Transmittance (blue) and SAS (black) spectra of 5S_1/2_(F = 2) − 5P_3/2_ (F′ = 1, 2, 3) transition as functions of pump-laser detuning frequency without coupling laser. (**b**) Normalized second-order auto-correlation functions *g*^(2)^(*τ*) for idler-mode scattered photons according to pump-laser detuning frequencies (resonance, 266 MHz, 532 MHz, and 1 GHz) which are estimated to be the optical depths of 4.3, 1.9, 0.23, and 0.002, respectively.

We investigated the temporal statistical properties of the SPs of the idler mode obtained via the 5S_1/2_ − 5P_3/2_ one-photon transition without the coupling field in the configuration shown in Fig. 1. Figure 2(a) shows the pump-laser transmittance spectrum for the 5S_1/2_(F = 2) − 5P_3/2_ (F′ = 1, 2, 3) transition in the Doppler-broadened warm atomic vapor of ^87^Rb atoms, where the vapor-cell temperature was set to 80 °C, the pump power was 0.5 mW, and the beam diameter was 1.2 mm. Under the condition of high optical depth (OD) of the atomic vapor, the pump light at near resonance with the 5S_1/2_(F = 2) − 5P_3/2_(F′ = 3) cycling transition was completely absorbed and scattered. However, when the pump-laser frequency was detuned from the on-resonance state, the scattering of the pump laser from the atoms was decreased because of its weak interaction with the atoms. In the case of far detuning of 1 GHz beyond the Doppler broadening, we were unable to observe the absorption of the pump laser in the isotope-enriched ^87^Rb vapor cell, even though the saturated absorption spectrum (SAS) of ^85^Rb was observed in the natural Rb vapor cell.

Figure 2(b) shows the normalized second-order auto-correlation functions *g*^(2)^(*τ*) for the spontaneously emitted photons of the idler mode according to the pump-laser detuning frequency, i.e., from resonance to 1 GHz, as shown by the red arrow heads of Fig. 2(b). The second-order correlation values *g*^(2)^(0) were measured as approximately 1.75, close to 2 of the bunched light g^(2)^(0). The photons due to the scattering of the pump laser interacting with the atoms clearly exhibit the photon statistical property of bunched light under any detuning condition. However, as the pump-field frequency was detuned from on-resonance to 1 GHz, the full width at half maximum (FWHM) of the *g*^(2)^(*τ*) spectrum was changed from 1.8 ns to 26 ns. Under the condition of on-resonance, the photons multi-scattered more than once were dominantly coupled to the optical fiber as a result of strong interactions with the atoms. Thus, the FWHM of the on-resonance *g*^(2)^(*τ*) spectrum was measured to be 1.8 ns, because of the Doppler broadening of the multi-SPs in the atomic vapor cell^14^. However, in the case of 1-GHz detuning, it dramatically changed to approximately 26 ns. This change occurred because of the presence of single-SPs due to weak atom-photon interactions under the condition of far detuning beyond the Doppler broadening. In the single- and multi-scattering coexistence regime, components of both the narrow and broad *g*^(2)^(*τ*) spectra are apparent at detuning frequencies of 266 MHz and 532 MHz, respectively. Therefore, we can elucidate the variance of the temporal statistical properties of the SPs of the 5S_1/2_ − 5P_3/2_ one-photon transition according to the pump-laser detuning frequency in the Doppler broadened atomic ensemble, by observing the single- and multi-scattering effects^14^.

### Scattered photons via two-photon transition in Doppler-broadened atomic medium

**Figure 3:**
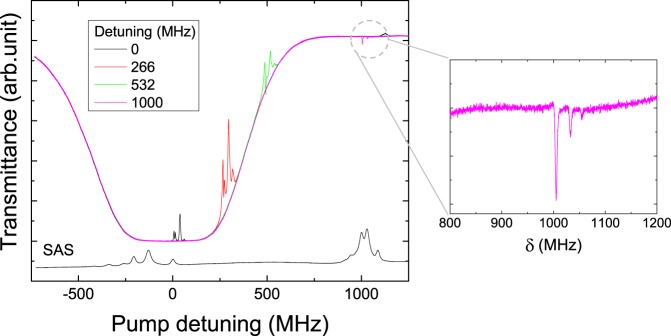
Transmittance spectra of 5S_1/2_(F = 2) − 5P_3/2_ (F′ = 1, 2, 3) transition as functions of pump-laser detuning frequency according to coupling-laser detuning frequencies (resonance, 266 MHz, 532 MHz, and 1 GHz).

**Figure 4:**
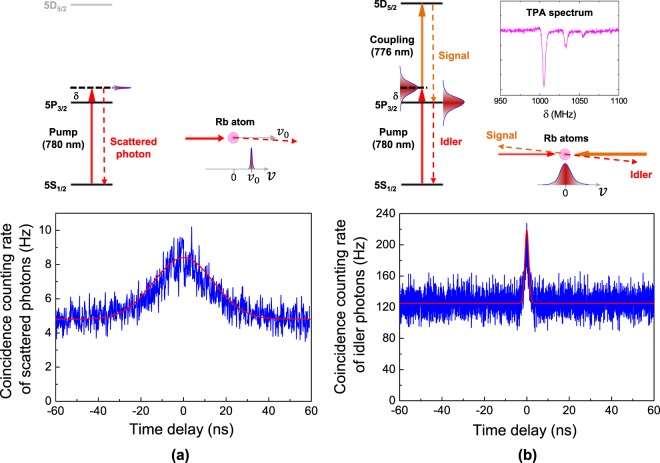
Comparing the two kinds of SPs obtained via one- and two-photon transitions. Temporal statistical properties of scattered photons according to (**a**) one-photon [5S_1/2_(F = 2) − 5P_3/2_(F′ = 3)] and (**b**) two-photon [5S_1/2_(F = 2) − 5P_3/2_(F′ = 3) − 5D_5/2_(F″ = 4)] transitions in ladder-type atomic system under single-scattering condition and 1-GHz detuning.

Figure 3 shows the transmittance spectra of the pump laser for the 5S_1/2_(F = 2) − 5P_3/2_ transition in the Doppler-broadened warm atomic vapor of ^87^Rb atoms, which was obtained when the pump and coupling lasers satisfied the two-photon resonance condition of the 5S_1/2_(F = 2) − 5P_3/2_  − 5D_5/2_ transition. The pump and coupling powers were 0.5 mW and 10 mW, respectively. The black, red, green, and magenta curves of Fig. 4 represent the transmittance spectra according to the coupling-laser detuning frequencies, which were detuned from the 5P_3/2_ (F′ = 3) −5D_5/2_(F″ = 4) transition to 0, 266 MHz, 536 MHz, and 1 GHz, respectively. Under the two-photon resonance, the dynamical variance in the two-photon coherence effects is apparent. In the case of far detuning of 1 GHz beyond the Doppler broadening, the two-photon absorption (TPA) spectrum of the 5D_5/2_(F″ = 2, 3, 4) state is clearly apparent, as shown in the magnified spectrum of Fig. 3 (inset).

In particular, to minimize the SPs due to the one-photon resonance but maximize the SPs due to the two-photon resonance, the pump laser was blue-detuned by approximately 1 GHz from the 5S_1/2_ (F = 2) − 5P_3/2_ (F′ = 3) transition. Subsequently, the coupling laser was red-detuned by approximately 1 GHz from the 5P_3/2_ (F′ = 3)−5D_5/2_ (F″ = 4) transition, to satisfy the conditions for the two-photon resonance of the 5S_1/2_ (F = 2) − 5P_3/2_(F′ = 3) − 5D_5/2_(F″ = 4) transition. Because of the two-photon coherence on the two-photon resonance, the signal photon of the 5P_3/2_(F′ = 3)−5D_5/2_(F″ = 4) transition and the idler photon of the 5S_1/2_(F = 2) − 5P_3/2_(F′ = 3) transition were emitted from the Doppler-broadened ladder-type atomic system.

However, it was unclear whether the temporal statistical properties of the SPs can vary based on the occurrence of either one- or two-photon transitions in the ladder-type atomic system of Fig. 1(a). To compare the two kinds of SPs obtained via one- and two-photon transitions from a Doppler-broadened warm atomic vapor for the 5S_1/2_ − 5P_3/2_ − 5D_5/2_ transition of ^87^Rb atoms, we investigated the coincidence events of the SPs obtained via one-photon (Fig. 4(a), without the coupling laser) and two-photon (Fig. 4(b), with the coupling laser) transitions under the single-scattering condition of 1-GHz detuning for a low OD value of 0.002.

Figure 4(a) shows the bunching events of the SPs of the one-photon transition according to coincidence counting in the HBT experiment. The average coincidence counting rate of the idler mode at *τ* = 0 was measured as approximately 8.5 Hz for a coincidence window of 5 ns and a detector dead time of ~50 ns. The SPs obtained via the one-photon transition of Fig. 4(a) were due to the 780-m/s velocity (*v*_0_) of the selected atoms interacting with the co-propagating pump laser. This behavior occurred because the co-propagating pump laser had Doppler shifts of −1 GHz, and atoms with *v*_0_ of near 780 m/s satisfy the resonance condition of the 5S_1/2_(F = 2) − 5P_3/2_ (F′ = 3) transition. The idler-mode SPs with a *θ*_t_ of 1.3° from the pump-laser propagation direction had Doppler shifts of +1 GHz × cos 1.3° from the resonance frequency of the 5S_1/2_(F = 2) − 5P_3/2_(F′ = 3) transition. For single scattering due to atom–photon interactions, the Doppler broadening (σ_s_) of the single-SPs can be expressed^14^ as

1 $${\sigma }_{s}=\,\sin \,{\theta }_{t}{\sigma }_{D}.$$

Here, σ_D_ is the Doppler width of the warm atomic vapor. When *θ*_t_ is very small, σ_s_ can be neglected. As shown in Fig. 4(a), the width of the temporal statistical spectrum of the SPs was measured to be approximately 26 ns, corresponding to the natural lifetime of the 5P_3/2_ state.

**Figure 5:**
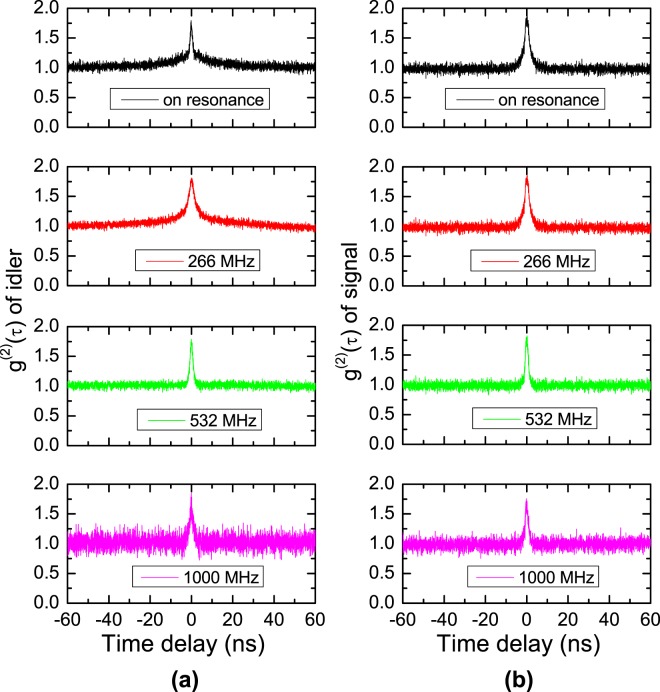
Normalized second-order auto-correlation functions *g*^(2)^(*τ*) for SPs of idler (**a**) and signal (**b**) modes according to pump- and coupling-laser detuning frequencies (resonance, 266 MHz, 532 MHz, and 1 GHz).

Figure 4(b) shows the HBT results for the SPs of the idler and signal modes obtained when the coupling laser was activated and both lasers satisfied the two-photon resonance condition of the 5S_1/2_(F = 2) − 5P_3/2_ (F′ = 3) − 5D_5/2_(F″ = 4) transition; the coupling power was 10 mW. Under the condition of 1-GHz detuning of the coupling laser, the TPA spectrum was apparent, as shown in the upper spectrum of Fig. 4(b) (see Fig. 3 for further details). We could simultaneously measure the SPs of the idler mode of the 5S_1/2_(F = 2) − 5P_3/2_(F′ = 3) transition and the signal mode of the 5P_3/2_(F′ = 3) − 5D_5/2_(F″ = 4) transition. The *g*^(2)^(*τ*) characteristics of the signal and idler photons were similar (see Fig. 5 for further details). Comparing the temporal statistical properties of the SPs obtained via two-photon transition (Fig. 4(b)) with the case of one-photon transition shown in Fig. 4(a), it is apparent that the average coincidence counting rate of the former at *τ* = 0 was more than 20 times higher than the latter, at approximately 200 Hz. Further, the width of the temporal statistical spectrum was dramatically narrower, i.e., by 15 times, at 1.8 ns. The single-scattering effect via single-photon transition for the SPs obtained via the two-photon transition of Fig. 4(b) is negligible, because the average coincidence counting rate obtained via two-photon transition was much higher than that of the SPs for the one-photon transition case.

The brightness and spectral narrowing were due to the coherent contributions from almost all the velocity classes in the Doppler-broadened atomic ensemble. Because the wavelength difference between the two lasers was very small, the two-photon resonant condition for the atoms interacting with the two counter-propagated lasers could be Doppler-free. The two-photon Doppler shift (*ω*_two_) for the 776-nm coupling laser (5P_3∕2_ − 5D_5∕2_ transition) and the 780-nm pump laser (5S_1∕2_ − 5P_3∕2_ transition) can be expressed as

2 $${\omega }_{two}=({k}_{p}-{k}_{C})\cdot v,$$

where *k*_p_ and *k*_C_ are the wave vectors of the pump and coupling lasers, respectively, and *v* is the atom velocity. The spectral width of the two-photon resonance spectrum was measured to be approximately 3 MHz, which was determined based on the linewidths and Rabi frequencies of both lasers. The value of *ω*_two_ was estimated to be 2.8 MHz at a velocity of 400 m/s. Therefore, the SPs obtained via the two-photon transition contributed to almost all the velocity classes of the atoms in the warm atomic vapor. The second-order auto-correlation functions *g*^(2)^(*τ*) expected in the Doppler width of the warm atomic vapor can be expressed^13,14^ as

3 $${g}^{(2)}(\tau )-1=\beta {e}^{-\frac{{\sigma }_{m}}{2\tau }},$$

where *σ*_m_ is the Doppler-broadened spectral width and *β* is the spatial coherence factor related to the correlation length at the detector position. In our experiment, the value of *β* was estimated to be close to 1, which is related to the correlation length *l*_c_ = λL/(πs) at the position of the detector. Here λ (780 nm), s (0.5 mm), and L (1.0 m) are the wavelength of the SP, radius of the emitted SP, and distance between light source and the fiber collimation lens, respectively. The main cause of the limitation of the values *g*^(2)^(0) is more dominant of the time jitter of the employed SPDs than the spatial coherence. The red curve in Fig. 4(b) shows the calculated result considering the convolution of the g^(2)^(*τ*) function and the time jitter (0.4 ns) of the SPD, which is close to the experimental results shown in Fig. 4(b). Therefore, we confirmed that the temporal statistical properties of the SPs can vary in accordance with the photons emitted from one- or two-photon transitions in the Doppler-broadened ladder-type atomic system.

Figure 5 shows the normalized second-order auto-correlation functions *g*^(2)^(*τ*) for the spontaneously emitted photons of the idler and signal modes for various pump- and coupling-laser detuning frequencies, corresponding to the four cases in Fig. 4. The second-order correlation values *g*^(2)^(0) were measured to be approximately 1.75 and the FWHMs of the TIC spectra were estimated to be approximately 2 ns. In the resonant case, the SPs obtained via one- and two-photon transitions were mixed and indistinguishable, because the FWHMs of both cases were similar. However, in each of the other three cases, the component of the broad *g*^(2)^(*τ*) spectrum due to the single-scattering effect via the one-photon transition is barely visible. Therefore, the two-photon transition dominantly contributed to the SPs of both the idler and signal modes.

## Conclusion

For the first time, we have experimentally demonstrated that the temporal statistical properties of the scattered photons can vary in accordance with the photons emitted from the one- or two-photon transitions in the Doppler-broadened warm atomic vapor with respect to the ladder-type two-photon transition routes between the hyperfine states in the 5S_1/2_ − 5P_3/2_ − 5D_5/2_ transition of ^87^Rb atoms. Because of the two-photon coherence on the two-photon resonance, the strongly correlated signal and idler photons were emitted from the warm atomic vapor. On the two-photon resonant condition for the atoms interacting with the two counter-propagated lasers, the correlated SPs obtained via the two-photon transition contributed to almost all the velocity classes of the atoms in the Doppler-broadened atomic ensemble. The two-photon Doppler shift for the 776-nm coupling laser and the 780-nm pump laser was estimated to be 2.8 MHz at a velocity of 400 m/s. This experimental results were elucidated as the velocity classes contributing to the SPs according to the transition routes in the Doppler-broadened ladder-type atomic ensemble. Our experimental results are expected to facilitate in understanding the dependence of the TIC of the SPs on the laser detuning and transition route in ladder-type atomic systems. Also, the spectral narrowing of the *g*^(2)^(*τ*) spectra of the correlated SPs generated via the two-photon transition is expected to relate to the superradiant effect in the Doppler-broaden ladder-type atomic scheme. We further believe that our results have very important implications for various fields in which the bunching properties of thermal light in quantum optics are of interest.

## References

[1] Mandel, L. & Wolf, E. *Optical coherence and quantum optics*, (Cambridge University Press, New York,1995).

[2] Loudon, R. *The Quantum Theory of Light*, *2*^*nd*^*3d*. (Oxford University Press, New York,1983).

[3] Strekalov DV, Sergienko AV, Klyshko DN, Shih YH (1995). Observation of two-photon “ghost” interference and diffraction. Phys. Rev. Lett..

[4] Scarcelli G, Valencia A, Shih YH (2004). Experimental study of the momentum correlation of a pseudo-thermal field in the photon-counting regime. Phys. Rev. A.

[5] Bache M (2006). Coherent imaging of a pure phase object with classical incoherent light. Phys. Rev. A.

[6] Peng T, Tamma V, Shih Y (2016). Experimental controlled-NOT gate simulation with thermal light. Sci. Rep..

[7] Ihn YS, Kim Y, Tamma V, Kim Y-H (2017). Observation of second-order temporal interference with thermal light: Interference beyond the coherence time. Phys. Rev. Lett..

[8] Morgan BL, Mandel L (1966). Measurement of photon bunching in a thermal light beam. Phys. Rev. Lett..

[9] Zhang D, Zhai Y-H, Wu L-A, Chen X-H (2005). Correlated two-photon imaging with true thermal light. Opt. Lett..

[10] Jurczak C (1995). Observation of intensity correlations in the fluorescence from laser cooled atoms. Opt. Comm..

[11] Bali S, Hoffmann D, Simán J, Walker T (1996). Measurements of intensity correlations of scattered light from laser-cooled atoms. Phys. Rev. A.

[12] Stites R, Beeler M, Feeney L, Kim S, Bali S (2004). Sensitive measurement of radiation trapping in cold-atom clouds by intensity correlation detection. Opt. Lett..

[13] Nakayama K, Yoshikawa Y, Matsumoto H, Torii Y, Kuga T (2010). Precise intensity correlation measurement for atomic resonance fluorescence from optical molasses. Opt. Express.

[14] Dussaux A (2016). Temporal intensity correlation of light scattered by a hot atomic vapor. Phys. Rev. A.

[15] Harris SE, Field JE, Imamoglu A (1990). Nonlinear optical processes using electromagnetically induced transparency. Phys. Rev. Lett..

[16] Petch JC, Keitel CH, Knight PL, Marangos JP (1996). Role of electromagnetically induced transparency in resonant four-wave-mixing schemes. Phys. Rev. A..

[17] Li Y-Q, Xiao M (1996). Enhancement of nondegenerate four-wave mixing based on electromagnetically induced transparency in rubidium atoms. Opt. Lett..

[18] Moon HS, Lee L, Kim JB (2008). Double resonance optical pumping effects in electromagnetically induced transparency. Opt. Express.

[19] Becerra FE, Willis RT, Rolston SL, Orozco LA (2008). Nondegenerate four-wave mixing in rubidium vapor: the diamond configuration. Phys. Rev. A.

[20] Willis RT, Becerra FE, Orozco LA, Rolston SL (2009). Four-wave mixing in the diamond configuration in an atomic vapor. Phys. Rev. A.

[21] Khadka U, Zheng H, Xiao M (2012). Four-wave-mixing between the upper excited states in a ladder-type atomic configuration. Opt. Express.

[22] Wen F (2014). Electromagnetically induced transparency-assisted four-wave mixing process in the diamond-type four-level atomic system. Opt. Materials.

[23] Chanelie’re T (2006). Quantum telecommunication based on atomic cascade transitions. Phys. Rev. Lett..

[24] Willis RT, Becerra FE, Orozco LA, Rolston SL (2011). Photon statistics and polarization correlation at telecommunications wavelengths from a warm atomic ensemble. Opt. Express.

[25] Ding D-S, Zhou Z-Y, Shi B-S, Zou X-B, Guo G-C (2012). Generation of non-classical correlated photon pairs via a ladder-type atomic configuration: theory and experiment. Opt. Express.

[26] Srivathsan B (2013). Narrow band source of transform-limited photon pairs via four-wave mixing in a cold atomic ensemble. Phys. Rev. Lett..

[27] Lee Y-S, Lee SM, Kim H, Moon HS (2016). Highly bright photon-pair generation in Doppler-broadened ladder-type atomic system. Opt. Express.

[28] Whiting DJ, Šibalić N, Keaveney J, Adams CS, Hughes IG (2017). Single-photon interference due to motion in an atomic collective excitation. Phys. Rev. Lett..

[29] Zugenmaier, M., Dideriksen, K. B., Sørensen, A. S., Albrecht, B. & Polzik, E. S., Long-lived non-classical correlations for scalable quantum repeaters at room temperature, *arXiv:1801*. *03286v1* (2018).

[30] Jeong, T., Lee, Y.-S., Park, J., Kim, H. & Moon, H. S., Quantum interference between autonomous single-photon sources from Doppler-broadened atomic ensemble. *Optica* **5** (2017).

[31] Kaczmarek, K. T. *et al*, A room-temperature noise-free quantum memory for broadband light. *arXiv:1704*.*00013v2* (2017).

[32] Hanbury Brown R, Twiss RQ (1956). Correlation between photons in two coherent beams of light. Nature.

[33] Kwiat PG (1995). New high-intensity source of polarization-entangled photon pairs. Phys. Rev. Lett..

[34] Sanaka K, Kawahara K, Kuga T (2001). New high-efficiency source of photon pairs for engineering quantum entanglement. Phys. Rev. Lett..

[35] Lee SM, Kim H, Cha M, Moon HS (2016). Polarization-entangled photon-pair source obtained via type-II non-collinear SPDC process with PPKTP crystal. Opt. Express.

[36] Förtsch M (2013). A versatile source of single photons for quantum information processing. Nature Comm..

[37] Rambach M, Nikolova A, Weinhold TJ, White AG (2016). Sub-megahertz linewidth single photon source. APL Photonics.

